# Interaction Dynamics and Comparative Biology of Two Hymenopterous Parasitoids of *Diaphorina citri* Kuwayama

**DOI:** 10.3390/insects17050444

**Published:** 2026-04-22

**Authors:** Ana María Restrepo-García, Jessika Alejandra Vanegas-Montoya, Maricarmen Sánchez-Borja, Jaime González-Cabrera, Alberto Soto-Giraldo

**Affiliations:** 1 Research Center, Asociación Colombiana de Estudios Vegetales, Carrera 15A No. 124-22, Bogotá 110110, Colombia; 2 Montelindo Farm, Faculty of Agricultural Sciences, Universidad de Caldas, Calle 65 No. 26-10, Manizales 170004, Colombia; jessikavanegas537@gmail.com; 3 Beneficial Insects of the North, Carretera Interejidal, Cd. Victoria, Tamaulipas 87260, Mexico; maricarmen.sanchez@insectosbeneficosdelnorte.mx; 4 Department of Biological Control (CNRCB), SENASICA, Km 1.5 Carr, Tecoman-Estacion FFCC, Colima 28110, Mexico; jgonz017@ucr.edu; 5 Faculty of Agricultural Sciences, Universidad de Caldas, Calle 65 No. 26-10, Manizales 170004, Colombia; alberto.soto@ucaldas.edu.co

**Keywords:** *Diaphorencyrtus aligarhensis*, *Tamarixia radiata*, Asian citrus psyllid, biological control, Huanglongbing

## Abstract

Huanglongbing (HLB) is the most serious disease affecting citrus production worldwide, and its management relies on controlling its vector, *Diaphorina citri*. Although *Tamarixia radiata* (Waterston) is widely used in biological control programmes, *Diaphorencyrtus aligarhensis* (Shafee et al.) has been proposed as a complementary agent; both species were reported in Colombia in 2016, yet their interaction dynamics under local conditions remain poorly understood. This study evaluated the development, morphometry, and interaction patterns of both parasitoids under controlled rearing conditions using a shared host population. Three treatments were established: non-parasitised nymphs, nymphs exposed to *D. aligarhensis*, and nymphs exposed to *T. radiata*, with parasitoids allowed to oviposit for 24 h. Developmental duration and morphometric traits were recorded daily. *T. radiata* completed its life cycle in less time (~11.7 days) than *D. aligarhensis* (~14.4 days) and than non-parasitized nymphs (~17 days). Morphometric traits were consistent with previously reported ranges, and this study provides the first detailed characterisation of the immature stages of *D. aligarhensis* in Colombia. Particularly, events of multiparasitoidism and superparasitoidism were documented under standardised conditions, demonstrating that both species can exploit the same host simultaneously. These findings offer new insights into parasitoid interaction dynamics and establish a biological foundation for developing integrated biological control strategies against *D. citri.*

## Simple Summary

Huanglongbing is a destructive disease that threatens citrus production worldwide and is spread by a small insect known as the Asian citrus psyllid. One of the most sustainable ways to manage this pest is by using natural enemies called parasitoids, which develop inside or on the insect and eventually kill it. Two parasitoid species frequently attack this pest, but they are usually studied separately, and little is known about how they behave when they occur together. In this study, we evaluated both species under controlled conditions and found that they can parasitize the host simultaneously. In some cases, more than one parasitoid developed within a single host, which may influence their effectiveness. We also compared their development rates and physical characteristics. These findings improve our understanding of how these natural enemies interact and provide useful information for designing more effective and sustainable pest control strategies, helping to reduce reliance on chemical insecticides in citrus production.

## 1. Introduction

One of the main phytosanitary threats to citrus production worldwide is the disease known as Huanglongbing (HLB); the associated economic losses are estimated to range between US$1 billion and US$15 billion annually, depending on the region and the level of crop impact [1,2]. The disease is caused by bacteria of the genus *Candidatus Liberibacter* spp., among which *Candidatus Liberibacter asiaticus* (CLas) stands out [3,4]. The main vector is *Diaphorina citri* Kuwayama (Hemiptera: Psyllidae), an insect that feeds on the phloem and facilitates the spread of the pathogen [5].

Despite advances in research on this disease, to date no viable cure has been achieved at a commercial scale [6]. This limitation, together with its rapid spread, has forced growers to adopt preventive management strategies, significantly increasing production costs [2]. Although broad-spectrum insecticides are currently the most cost-effective management strategy, they represent a constant and intense threat to the environment, producers, and consumers; therefore, sustainable and environmentally friendly alternatives are needed [1,7]. In response to this threat, biological control offers a long-term management solution based on the conservation of local natural enemies, the introduction of new beneficial agents, the permanent establishment of their populations, and the mass rearing with periodic or seasonal releases of these organisms [8,9].

Parasitoids stand out for their high specificity, as they selectively attack pest species and allow their populations to be reduced without harming non-target organisms [10,11]. Among them, *Tamarixia radiata* (Waterston) (Hymenoptera: Eulophidae) and *Diaphorencyrtus aligarhensis* (Shafee et al.) (Hymenoptera: Encyrtidae) have been identified as the main natural enemies of *D. citri* and have been used in classical biological control programmes worldwide [12,13,14].

*T. radiata* is an ectoparasitoid that parasitises third- to fifth-instar nymphs and completes its development externally on the host [12]. At temperatures of 25–30 °C it exhibits its highest efficacy, with optimal performance at 26 °C [15,16]. Under these conditions, parasitism levels ranging from 2% to 91% have been recorded, depending on vector density and microclimatic conditions [17,18]. In contrast, *D. aligarhensis* is an endoparasitoid that oviposits in second- to fourth-instar nymphs and completes its life cycle within the host and has reached parasitism rates of up to 17% under favourable conditions [12,19,20]. However, in countries such as Colombia and Ecuador, these rates are usually lower, ranging from 0.3% to 1.0% under field conditions [20,21]. Milosavljević et al. [22] suggest that *D. aligarhensis* may tolerate extreme thermal conditions, such as temperatures above 35 °C for more than 11 h; consequently, it survives longer at constant intermediate temperatures and achieves more stable populations.

Although several attempts have been made to establish *D. aligarhensis* in California using specimens originating from Pakistan, no mass releases of this species comparable to those conducted with *T. radiata* have been documented [23,24], and its natural presence in citrus orchards in the region has also not been precisely described. In Colombia, both genera of parasitoids have been reported coexisting in the same citrus-growing area [25,26]; however, under local conditions, a knowledge gap persists regarding key aspects of their biological performance, including development time, morphometry of immature stages, diagnostic traits of parasitism, and possible interactions, especially in the case of *D. aligarhensis*. In particular, the absence of studies evaluating both parasitoid species under the same experimental conditions limits the understanding of their interaction dynamics, including multiparasitoidism and superparasitoidism, which may be critical for their combined use in biological control programmes. This gap also hinders the assessment of the contribution of *D. aligarhensis* and the feasibility of integrating both species into complementary management strategies.

In this context, the present study aimed to biologically and morphologically characterise *T. radiata* and *D. aligarhensis* parasitising *D. citri* in an experimental rearing programme in Colombia, through the evaluation of development time, the morphometry of immature stages, the diagnostic traits of parasitism, and the occurrence of multiparasitoidism and superparasitoidism.

## 2. Materials and Methods

### 2.1. Study Site and Rearing Conditions

The study was conducted at Montelindo Farm of the University of Caldas (latitude 5.075097, longitude −75.672948). The rearing system comprised three simultaneous components: 1. Maintenance of the host plant, 2. Reproduction of the Asian citrus psyllid, and 3. Multiplication of the parasitoids. Each component was developed in greenhouse-type structures equipped with anti-aphid mesh and a double-door access system. For this purpose, the protocol of the National Reference Center for Biological Control [27] was adapted, with specific modifications in host plant management, parasitoid release density, and exposure time, adjusted to the local rearing conditions of the experimental system.

*Murraya paniculata* (Linnaeus) Jack plants 15 to 20 cm in height were obtained from a commercial nursery. Each plant was transplanted into No. 26 pots (≈5 kg of substrate) containing a 3:1 mixture of loamy soil and organic matter. When plants reached 30–40 cm in height, the apical portion (≈5 cm) was cut to stimulate branching. Irrigation was performed manually every two days, or earlier if environmental conditions required it. At the end of each production cycle, plants were pruned and fertilised with 150 g of organic matter and set aside for recovery. During the recovery phase, pest control was also carried out through foliar applications of garlic and chilli extract and/or 5% soap solution. Two days after application, plants were sprayed with water to remove residues.

The production of *D. citri* was established on two-year-old *M. paniculata* plants planted directly in the soil. The initial psyllid specimens were collected from citrus crops within the same experimental farm, located in an area declared free of HLB disease [28]. Adults were captured using an entomological aspirator, transferred to the rearing area, and released en masse. The plants were pruned sequentially every three months to promote the natural migration of adults towards new shoots. After each pruning, 200 g of organic matter per plant was applied to stimulate shoot emergence.

The production of parasitoids was carried out in entomological cages measuring 70 × 70 × 70 cm, constructed with PVC pipe supports and covered with muslin fabric, with a rubber floor, plastic door, and U-shaped zipper closure. In each cage, four *M. paniculata* plants with shoots 5–10 cm in length were introduced, and 300 adults of *D. citri* were released and maintained for 7 days. Subsequently, the adults were removed with the aid of an entomological aspirator, leaving the plants with only eggs and early-instar nymphs.

The first specimens of *D. aligarhensis* were obtained as mummies on *Citrus latifolia* (Tahiti lime) at the experimental farm. The species was confirmed using previously reported taxonomic keys [29,30], as well as by the statement that the report of *Diaphorencyrtus* sp. made by Arias-Ortega et al. [25] from the department of Caldas corresponds to *D. aligarhensis* [20]. Once the adults of *D. citri* were removed, 100 adults of the parasitoid were released into each cage, taking advantage of the presence of early instars. The insects remained in contact with the host for 7–10 days; subsequently, the specimens were collected with an entomological aspirator to allow the development of the mummies.

The initial colony of *T. radiata* was established from 100 individuals obtained from an established laboratory colony maintained under institutional conditions. Seven days after the removal of *D. citri* adults, and after verifying the presence of third- and fourth-instar nymphs, 150 adults of *T. radiata* were released at a 2:1 ratio (female ♀: male ♂). The parasitoids remained in contact with the host for 7–10 days and were subsequently removed using an entomological aspirator to allow the development of the mummies. Both parasitoid species were maintained in separate cages, each provided with strips of absorbent paper impregnated with pure honey as a food source for the adults. The colonies were maintained at 27 ± 10 °C, 76 ± 20% relative humidity, and a photoperiod of 12 ± 1 h of light.

### 2.2. Experimental Design

To characterize the biology and morphology of the arthropods in the experimental rearing system, a completely randomized experimental design with three treatments was employed: T1 = control without parasitoid (*D. citri*), T2 = release of *D. aligarhensis*, and T3 = release of *T. radiata*. Each treatment had two replicates, represented by two entomological cages, each containing four *M. paniculata* plants. Initially, 400 adults of *D. citri* were introduced into each experimental unit and left in contact for 24 h to obtain a homogeneous host population. In T2, when the population reached second- and third-instar nymphs, 100 adults of *D. aligarhensis* were released. In T3, upon observing third- and fourth-instar nymphs, 150 adults of *T. radiata* were released at a 2♀:1♂ ratio. In both treatments, the parasitoids remained in contact with the host for 24 h and were subsequently removed using an entomological aspirator. In the cages corresponding to the control treatment (T1), the population of *D. citri* continued its cycle without exposure to parasitoids.

### 2.3. Development Monitoring and Morphometric Measurements

Every 24 h, random destructive sampling was conducted in the cages of each treatment. In each sampling event, 15 specimens per treatment were selected and evaluated to record their developmental stage and to perform morphometric measurements of length and width using a stereomicroscope stereomicroscope BS3035B3 (BestScope, Beijing, China) equipped with a micrometric eyepiece. This procedure was carried out daily until the emergence of the adults corresponding to each treatment. In the case of *D. aligarhensis*, dissection of parasitized nymphs was required to expose the immature stages of the endoparasitoid and allow their observation and measurement. Additionally, the marks or diagnostic traits of parasitoidism observed during the development of each species were recorded, as well as the duration of development from egg to adult.

Developmental time was defined as the duration required for the last observed individual in each treatment to complete development to the adult stage; therefore, variability measures such as standard deviation were not calculated for this parameter.

For image analysis and scale representation, digital images were calibrated in ImageJ.JS v0.6.0 (https://ij.imjoy.io/, accessed on 11 April 2026) using reference measurements previously obtained with the micrometric eyepiece. This allowed the conversion of pixel-based measurements into real units and the generation of accurate scale bars for all images.

### 2.4. Statistical Analysis

The obtained data were tested for the assumption of normality using the Shapiro–Wilk test and for homogeneity of variances using Levene’s test. The duration of the life cycle was compared among treatments using analysis of variance (ANOVA) with a significance level of 0.05. When significant differences were detected, Duncan’s multiple range test was applied. All analyses were performed using IBM SPSS^®^ Statistics Version 20.

## 3. Results

### 3.1. Development Time of the Species

The development time from egg to adult differed significantly among treatments (ANOVA: *F* = 67.486; *p* < 0.001). *T. radiata* completed its development in the shortest time (11.73 days), followed by *D. aligarhensis* (14.4 days), whereas the control treatment (without exposure to parasitoids) showed the longest duration (17 days), based on the time required for the last observed individual to reach adulthood in each treatment.

### 3.2. Morphometric Characterization of Developmental Stages

The average values of length, width, and duration of the different developmental stages of the species are presented in Table 1. In general, the three arthropods showed consistent and progressive size variation across their ontogenetic stages, with a clear increase in dimensions throughout development. In *D. citri*, the largest dimensions were recorded in the advanced nymphal stages and in the adult stage. In *T. radiata*, the differences among egg, larva, prepupa, pupa, and adult clearly defined the developmental sequence. Similarly, in *D. aligarhensis*, gradual changes in length and width were observed from egg to adult, facilitating the differentiation of immature stages under rearing conditions.

### 3.3. Monitoring the Internal Development of D. aligarhensis

**Figure 1:**
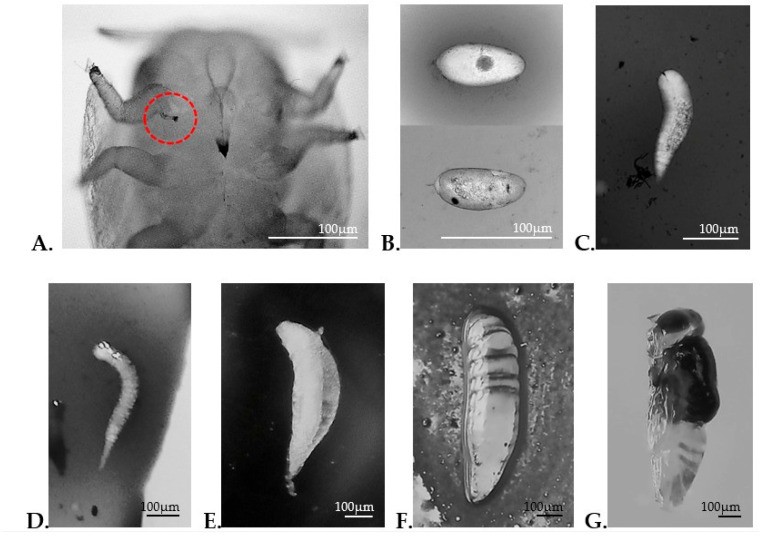
Developmental stages of *D. aligarhensis*. (**A**) Parasitism mark on the ventral region of *D. citri* (red circle); (**B**) egg, 12 h after parasitism (upper), 48 h after parasitism (lower); (**C**) first instar larva; (**D**) second instar larva; (**E**) third instar larva; (**F**) fourth instar larva; (**G**) pupa.

Destructive sampling allowed the precise observation and recording of the internal stages of *D. aligarhensis*, through dissections performed on nymphs exhibiting signs of parasitism (Figure 1A). This procedure enabled confirmation of parasitoid presence and the documentation of the complete morphometric sequence of its developmental stages from egg to adult (Figure 1B–G). This approach facilitated the accurate identification of developmental stages that are not externally visible in endoparasitoids.

### 3.4. Diagnostic Traits of Parasitism and Mummy Morphology

**Figure 2:**
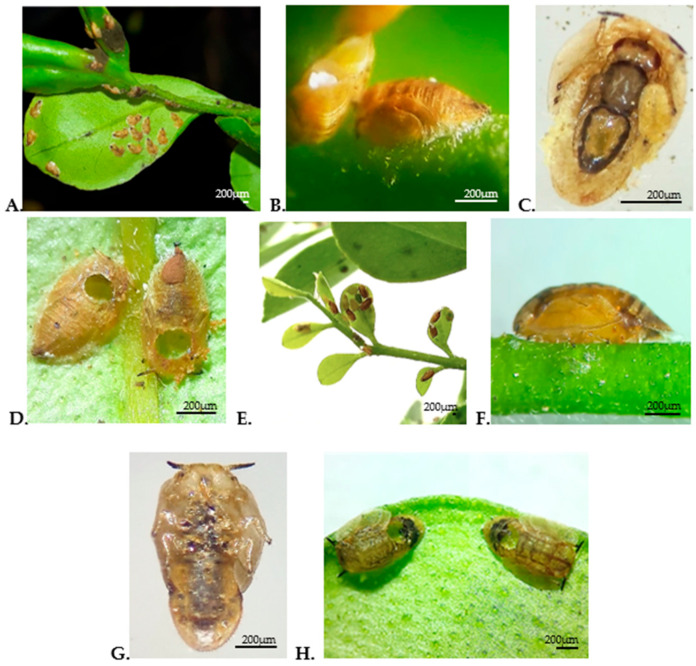
Morphology of *D. citri* mummies parasitized by *T. radiata* and *D. aligarhensis*. (**A**) Mature shoot of *M. paniculata* with nymphs parasitized by *T. radiata*; (**B**) *T. radiata* mummies attached to plant tissue; (**C**) ventral view of parasitized nymphs with visible development of *T. radiata*; (**D**) adult emergence holes of *T. radiata*; (**E**) tender shoot with nymphs parasitized by *D. aligarhensis*; (**F**) *D. aligarhensis* mummies attached to plant tissue; (**G**) ventral view of parasitized nymphs with visible development of *D. aligarhensis*; (**H**) adult emergence holes of *D. aligarhensis*.

Clear differences were observed between both parasitoid species in the traits associated with parasitism and in mummy morphology. *T. radiata* formed most mummies on mature branches of *M. paniculata* (Figure 2A); these showed a brown coloration, a flattened appearance, and the presence of meconium in the posterior region (Figure 2B), as well as external development of the parasitoid attached to the ventral surface of the *D. citri* nymph (Figure 2C). The adult emergence hole was located in the anterior region of the mummy (Figure 2D).

In contrast, *D. aligarhensis* generated most of its mummies on tender shoots of the plants (Figure 2E). These were characterized by a brown coloration, a hemispherical shape, and absence of meconium (Figure 2F). Additionally, no visible parasitoid structures were observed on the ventral surface of the parasitized nymph (Figure 2G), and the adult emergence hole was located at the lower end of the mummy (Figure 2H).

### 3.5. Evidence of Multiparasitoidism, Superparasitoidism, and Host Feeding

**Figure 3:**
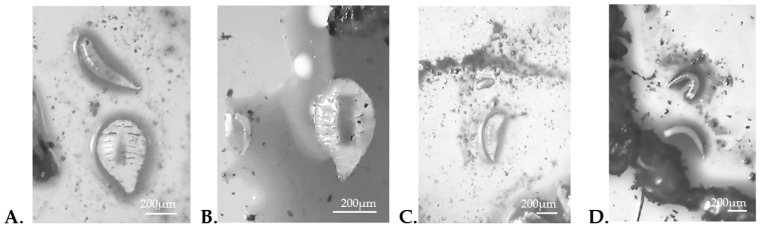
Evidence of multiparasitoidism and superparasitoidism. (**A**) Third instar larva of *D. aligarhensis* (upper), third instar larva of *T. radiata* (lower); (**B**) second instar larva of *D. aligarhensis* (left), third instar larva of *T. radiata* (right); (**C**) egg of *T. radiata* (upper), second instar larva of *D. aligarhensis* (lower); (**D**) two second instar larvae of *D. aligarhensis*.

Events of multiparasitoidism and superparasitoidism were recorded in *D. citri*. In multiparasitoidism events, nymphs were identified that simultaneously exhibited a parasitism mark compatible with *D. aligarhensis* and external larval development characteristic of *T. radiata* (Figure 3A–C). These observations indicate that both parasitoid species were able to exploit the same host individual concurrently. In contrast, superparasitoidism was observed mainly in *D. aligarhensis*, as nymphs showing multiple parasitism marks were detected and, after dissections, more than one developing individual was observed within a single host (Figure 3D).

During the observations, evidence of host feeding was recorded in both parasitoid species on first- and second-instar nymphs. This behavior was manifested by small dorsal scars associated with ovipositor insertion and the subsequent ingestion of haemolymph by adults.

## 4. Discussion

### 4.1. Differences in Development Associated with Parasitoid Strategy

The development time of *T. radiata* and *D. aligarhensis* in their host *D. citri* differed among treatments, reflecting differences in their parasitism strategies and host-stage preferences. In *T. radiata*, development occurs on N4 or N5 nymphs, whose stages exhibit longer durations, thereby broadening the parasitism window. In addition, as in an idiobiont parasitoid, the host nymph becomes paralyzed immediately after oviposition, interrupting its development [12,14]. This condition could favour the completion of the larval cycle on an immobile host, without depending on host development, which may explain its shorter life cycle and its recognised efficiency in biological control programmes [15,24].

In contrast, the development time of *D. aligarhensis* was longer and its larval phase corresponded to a koinobiont strategy, in which the host continues its development while the parasitoid matures internally [12]. This synchrony with the natural development of *D. citri* may explain both the prolonged development of the parasitoid and its comparable size to non-parasitised hosts, as it allows the progressive use of host resources; however, it could also limit its efficiency in the field, since the preferred nymphal stages are of short duration, reducing the temporal window available for parasitism. Additionally, related studies mention that a longer parasitism process may be less effective in environments where rapid control of host populations is required [14].

### 4.2. Biological Scope of Morphometric Characterization

The morphometry of parasitoids is crucial for understanding their development and the relationships among the sizes of their morphological traits [31], which may ultimately influence their performance as biological control agents. The morphometric measurements of *D. citri* obtained in this study were, in general terms, similar to those reported by Tsai et al. [32] and Fonseca et al. [33]; however, partial differences were observed with respect to García et al. [34], particularly in the N3 and N4 stages. These discrepancies may be associated with variations in rearing conditions, the plant host used, or methodological differences in the measurement process.

For *T. radiata*, the dimensions recorded for the different developmental stages showed some variation compared with those described by Mann and Stelinski [35], especially in fourth-instar larvae and adults. Nevertheless, the observed values remain within the ranges compatible with those reported for the species and support the use of morphometric characterisation as a complementary criterion for the identification of its immature stages.

In the case of *D. aligarhensis*, this study constitutes, to the best of current knowledge, the first morphometric report in Colombia of its immature stages under controlled conditions. The measurements obtained fell within the ranges reported by Rohrig et al. [30], supporting the consistency of the results and expanding the available knowledge for this species in the country. In addition, the use of directed dissections on nymphs showing signs of parasitism proved to be an effective approach for confirming parasitism and tracking the internal development of the parasitoid, particularly in studies where adult emergence cannot be monitored immediately.

For their part, the differences observed in the location, morphology, and emergence pattern of the mummies allow both parasitoid species to be clearly distinguished during rearing and monitoring. These differences are consistent with those described in various studies that reflect the particularities of their ectoparasitic and endoparasitoid modes of development, respectively [20,22,36]. Beyond its descriptive value, this information provides a standardised baseline for comparative studies and supports the evaluation of parasitoid performance under controlled conditions.

### 4.3. Implications of Multiparasitoidism, Superparasitoidism, and Host Feeding in Biological Control

The detection of multiparasitoidism and superparasitoidism provides evidence of potential scenarios of intra- and interspecific competition on *D. citri*. In the case of multiparasitoidism, the simultaneous presence of both parasitoid species within a single host indicates that these species can exploit the same host resource under overlapping temporal conditions. This suggests that host discrimination mechanisms may not completely prevent interspecific oviposition [13,37].

Previous studies have extensively documented the biology, ecology, and effectiveness of parasitoids associated with *D. citri*, particularly in the context of classical biological control and host–parasitoid interactions under different environmental conditions [30,38,39]. However, these studies have primarily focused on individual species, and limited attention has been given to the interaction dynamics between co-occurring parasitoids within the same host population. In this context, the present findings provide empirical evidence that both parasitoid species can exploit the same host, highlighting the occurrence of overlapping resource use and potential competitive interactions under controlled conditions, and contributing to a better understanding of their potential coexistence and implications for biological control strategies.

On the other hand, the superparasitoidism recorded in *D. aligarhensis* confirms that the same host can receive multiple ovipositions, as has also been described by Rohrig et al. [23]. As in the present study, it has not been possible to determine whether more than one individual can successfully complete development within a single host, highlighting the need for further evaluation of the outcomes of intra-specific competition.

From an applied perspective, these interaction patterns may influence the effectiveness of biological control strategies, particularly in systems where both parasitoid species coexist. In this context, the lack of synchronisation between species, as well as potential competitive interactions, may limit the expected additive or synergistic effects of combined releases.

Finally, the evidence of host feeding in both parasitoids suggests that, in addition to the direct effect of parasitism, these species may contribute to host mortality through the consumption of haemolymph from early-instar nymphs. This behavior, previously described by Chien [40] and Wan et al. [14], could increase the regulatory impact of both species on *D. citri* populations, while also providing resources for the reproductive maturation of females.

### 4.4. Relevance of Interaction Dynamics in the Context of Biological Control Programmes

From an applied perspective, these interaction patterns can be better understood when considered in the context of existing biological control programmes. In California, *T. radiata* has successfully established since its release and has been widely used in urban biological control programmes, showing consistent parasitism rates and contributing to reductions in *D. citri* populations [41,42]. In Texas, similar trends have been observed, where the establishment of *T. radiata* has been associated with significant decreases in psyllid populations following its release [43]. In contrast, *D. aligarhensis* has shown limited establishment in California, with low parasitism rates, and has not achieved consistent establishment in Florida despite repeated introduction efforts [23,42].

These contrasting outcomes highlight the complexity of factors influencing parasitoid performance in field conditions, including climatic suitability, host availability, interspecific competition, and release strategies [13,44]. Previous studies have also reported stage-specific host preferences and potential competitive interactions between *T. radiata* and *D. aligarhensis*, which may influence their effectiveness when both species are present [36].

In this context, particularly considering the natural co-occurrence of both species in Colombian citrus systems, the present study provides comparative biological evidence under controlled conditions that help interpret these field patterns. The interaction patterns observed in this study indicate that both species can exploit the same host individual, suggesting that interspecific interactions may influence their joint performance.

These findings offer a mechanistic basis for understanding the variability observed in applied programmes and underscore the importance of integrating species-specific biology and interaction dynamics into biological control planning. The limited establishment of *D. aligarhensis* in regions where *T. radiata* is dominant may be partially explained by competitive interactions at the host level, as suggested by the patterns documented here.

These findings emphasise the need for improved mass-rearing and release strategies that consider not only individual species performance but also their potential interactions. This is particularly relevant given the current limitations in the standardisation of rearing protocols for *D. aligarhensis*, in contrast to *T. radiata*, which has been more successfully integrated into biological control programmes [45].

## 5. Conclusions

The results demonstrate that *T. radiata* and *D. aligarhensis* exhibit complementary biological attributes on *D. citri*, differing in their development time, parasitism pattern, and use of host nymphal stages. Importantly, both species were able to exploit the same host, revealing interaction mechanisms such as multiparasitoidism and superparasitoidism under controlled conditions. These findings highlight their potential value in biological control and integrated pest management strategies against this vector. In addition, the morphometric characterization of the immature stages, particularly in *D. aligarhensis*, expands the knowledge available in Colombia and provides useful criteria for its recognition and monitoring in rearing programmes.

Overall, this study establishes a standardised biological framework for evaluating interactions between parasitoid species and supports the development of more effective biological control strategies. These findings provide a basis for improving mass-rearing approaches and for further validation of parasitoid performance under semi-field and field conditions.

## Figures and Tables

**Table 1:** Mean length, width, and developmental time of each stage of *D. citri*, *D. aligarhensis*, and *T. radiata* under controlled conditions.

*D. citri*	H	N1	N2	N3	N4	N5	A	
Length (mm)	0.29 ± 0.05	0.34 ± 0.04	0.49 ± 0.04	0.69 ± 0.05	0.98 ± 0.04	1.53 ± 0.20	2.25 ± 0.25	
Width (mm)	0.14 ± 0.01	0.17 ± 0.03	0.30 ± 0.02	0.47 ± 0.03	0.78 ± 0.03	1.15 ± 0.15	0.74 ± 0.08	
Time (days)	4.00	3.60	1.67	1.53	3.07	3.13	--	
*D. aligarhensis*	H	L1	L2	L3	L4/Prepupa	Pupa	A	
Length (mm)	0.13 ± 0.02	0.31 ± 0.09	0.70 ± 0.09	0.95 ± 0.15	1.20 ± 0.01	1.36 ± 0.11	1.30 ± 0.03	
Width (mm)	0.07 ± 0.01	0.08 ± 0.01	0.18 ± 0.08	0.23 ± 0.04	0.44 ± 0.50	0.50 ± 0.10	0.46 ± 0.04	
Time (days)	2.67	1.33	1.47	2.53	1.53	4.87	--	
*T. radiata*	H	L1	L2	L3	L4	Prepupa	Pupa	A
Length (mm)	0.17 ± 0.03	0.28 ± 0.01	0.38 ± 0.02	0.75 ± 0.06	0.94 ± 0.04	1.12 ± 0.02	1.31 ± 0.05	1.18 ± 0.1
Width (mm)	0.08 ± 0.02	0.10 ± 0.01	0.27 ± 0.02	0.44 ± 0.03	0.52 ± 0.03	0.60 ± 0.01	0.59 ± 0.03	0.36 ± 0.05
Time (days)	2.00	0.87	0.93	1.20	1.53	0.87	4.33	--

Note: Values are presented as mean ± standard deviation. H, egg; N1, first-instar nymph; N2, second-instar nymph; N3, third-instar nymph; N4, fourth-instar nymph; N5, fifth-instar nymph; L1, first-instar larva; L2, second-instar larva; L3, third-instar larva; L4, fourth-instar larva; A, adult.

## Data Availability

The original contributions presented in this study are included in the article. Further inquiries can be directed to the corresponding author.
